# Laboratory Study on the Stability of Large-Size Graded Crushed Stone under Cyclic Rotating Axial Compression

**DOI:** 10.3390/ma14071584

**Published:** 2021-03-24

**Authors:** Bo Tan, Tao Yang, Heying Qin, Qi Liu

**Affiliations:** 1 College of Civil and Architecture Engineering, Guilin University of Technology, Guilin 541004, China; 2000015@glut.edu.cn (B.T.); 1020180360@glut.edu.cn (T.Y.); 15264714363@163.com (Q.L.); 2 Guangxi Key Laboratory of New Energy and Building Energy Saving, Guilin 541004, China

**Keywords:** large-size graded crushed stone, cyclic rotating axial compression, shakedown theory, cumulative axial strain, long-term stability, critical load

## Abstract

In this paper, the stability of large-size graded crushed stone used for road base or cushioning under repeated load is investigated. Using an in-house developed device, large-size crushed stone mix was compacted and molded by the vibration and rotary compaction method. Cyclic rotating axial compression was applied, and the shakedown theory was used to study the cumulative deformation of the large-size crushed stone specimens. The effects of gradation parameters on the cumulative strain and stability behavior were analyzed, and the critical stability and failure loads were determined according to the shakedown theory. The test results indicate that there are three obvious instability behavior stages of large-size graded crushed stone under cyclic rotating axial compression: elastic stability, plastic creep, and incremental plastic failure. Large-size graded crushed stone has a higher critical stability load stiffness than conventional-size graded crushed stone. The critical shakedown load of the specimen is mainly affected by the skeleton structure performance, and the critical failure load by the properties of the crushed stone material. Increasing the content and compactness of large-size crushed stone in the specimen can improve the stiffness and stability performance, and to achieve improvements, the content of large-size crushed stone should be controlled between 22% and 26%. The critical shakedown load increases with the increase in the California bearing ratio (CBR) value, while, on the other hand, the CBR value has little relationship with the critical failure load.

## 1. Introduction

Large-size graded crushed stone, which is a typical elastic–plastic granular material [1,2,3,4,5,6], finds its applications as road base, highway cushion, and as airstrips. Its nominal maximum particle size is generally between 25 and 63 mm. The large-size graded crushed stone used in engineering applications has better stiffness, load bearing capacity, and pressure stability than the conventional-size graded crushed stone [7,8,9,10]. The deformation of road base caused by long-term vehicular cyclic loads is generally divided into elastic and plastic deformation. Elastic deformation has little fluctuation and can be recovered, while plastic deformation will gradually accumulate and can seriously affect the long-term stability of the road structure. However, there are only limited applications of large-size graded crushed stone in inland and foreign projects. The reason is that the performance and stability of large-size graded crushed stone are not fully understood.

Existing experimental studies on the stability of road base were mainly focused on conventional-size graded crushed stone or other granular materials. Cyclic loading was applied to the specimens by a repeated load triaxial (RLT) or material testing system (MTS) tester manufactured by the American MTS company to observe their deformations, and the stability was examined [11,12]. In recent years, numerous researchers have advanced understanding by adopting the shakedown theory to analyze the deformation behavior of granular materials under cyclic loading. Werkmeister [13,14] analyzed the stability of granular materials under cyclic loading using RLT tests and evaluated the deformation behavior of granular materials by shakedown theory. It was noted that the deformations of granular materials under cyclic loading can be divided into three stable behaviors: stable plastic behavior, plastic creep behavior, and incremental failure behavior, and the corresponding classification criteria and analysis methods were proposed. Xiao [15] performed both constant and variable confining pressure tests on the granular materials by using an advanced cyclic loading RLT device. It was found that the dynamic stress states can model the moving wheel loads effectively and shakedown theory can accurately describe the deformation behavior of granular materials. Several studies [16,17,18,19,20] also indicated that the critical shakedown load of granular materials under cyclic loading increased linearly with the increase in material yield stress. Moreover, the stability behavior of granular materials under cyclic loading is closely related to the confining pressure, dynamic stress amplitude, and fine aggregate content.

The cyclic loading was exerted by a traditional RLT tester designed for testing granular materials under axial and confining pressures and an MTS tester used in previous studies to test the deformation behavior of granular materials under cyclic loading. The actual compaction at a road construction site is usually due to the simultaneous action of axial pressure and shear stress [12,15]. Therefore, the combined effect of axial pressure and shear stress should be considered when studying the deformation behavior of granular materials under cyclic loading.

In this paper, adopting the rotation compression used in the design method of asphalt mixtures such as Superpave, cyclic rotating axial compression tests were conducted to study the deformations of the large-sized graded crushed stone under cyclic loading using a novel testing device developed in-house to achieve the simultaneous action of axial pressure and shear stress. The stability behavior was then evaluated by the shakedown theory. The step-by-step filling method [21] and the I method [22] were applied to design the gradation of large-size crushed stone mix, and the influences of gradation parameters on the mechanical properties and stability of the mix were analyzed. A CBR (California bearing ratio) test was carried out to investigate the relationship between the CBR values and the stability and critical shakedown load. This research provides a theoretical basis for the optimal design of large-sized graded crushed stone for engineering applications.

## 2. Shakedown Theory

### 2.1. Principles of Shakedown Theory

The shakedown theory, also known as the structural stability theory, was initially developed to study the deformation characteristics of metallic structures with clear elastic–plastic behavior and subject to the joint action of temperature and loads [12]. Subsequently, the theory was adopted by Sharp et al. for studying pavement material structure [23,24]. Based on the numerous previous investigations and reviews, it is considered that materials or structures generally exhibit three types of unstable behaviors, i.e., elastic stability, plastic creep, and incremental plastic failure, and two critical loads, i.e., critical shakedown load and critical failure load [25,26].

It is generally believed that when the stresses in the road material or structure are lower than a certain value, the strain increase rate becomes smaller with the increase in the number of load cycles, and the strain tends to stabilize. The corresponding stress is generally defined as the critical shakedown load, which refers to a threshold value of the loading stress when the material reaches the elastic shakedown state. If the cyclic loading is smaller than the critical shakedown load, the response of the structure is elastic. The final plastic deformations tend to be a stable and structural failure due to excessive accumulation of plasticity will not occur. The instability of the specimen under this stress level is called the shakedown state [14]. When the stresses in the road material are greater than a certain value, the stable state is destroyed, the strain rate shows no sign of decreasing, may even increase, and accumulates rapidly. The stress when this happens is defined as the critical failure load. The pavement material or structure will be destroyed when the load is greater than the critical failure load, and the specimen will find itself in the state of incremental plastic failure. When the load is greater than the critical shakedown load but less than the critical failure load, the specimen is in the plastic creep state [12], the strain of the specimen in this state increases slowly with the increase in the number of load cycles, and the strain rate stays small.

The graded crushed stone mixture has a clear skeleton structure and obvious cumulative deformation behavior when subjected to cyclic loading. In practical engineering applications, cumulative deformation will occur when the road is subjected to excessive cyclic loading, and the internal structure will be destroyed when the accumulated deformation becomes excessive. This will cause cracking between the road base and surface layer, which will affect the overall stability of the road structure [27]. Therefore, to study the stability of the large-sized graded crushed stone mixture, it is necessary to analyze its critical shakedown load, critical failure load, and deformation relationships. The optimal gradation design method suitable for the large-sized crushed stone can be developed by analyzing the relationship between the critical loads, deformations, and gradation type of crushed stone.

### 2.2. Stability Test and Behavior Evaluation

**Figure 1:**
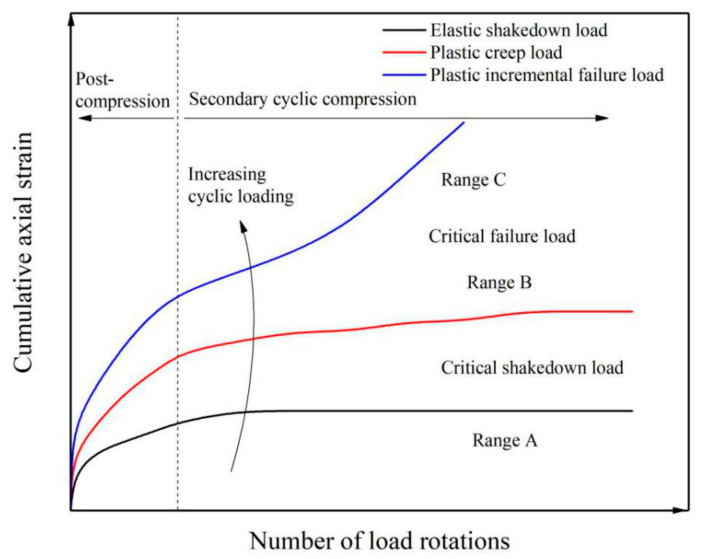
Deformation behavior of granular material under cyclic loading.

There are two main testing methods for studying the structural stability of granular materials: RLT test and MTS test. Both methods can perform triaxial cyclic loading tests, while the MTS testing machine can also perform axial cyclic loading tests. Using the two experimental methods, researchers have achieved significant scientific progress. In reference [28], the state of a granular material subjected to cyclic loads was divided into three ranges: range A (plastic shakedown), range B (plastic creep), and range C (incremental collapse). The critical shakedown load is at the transition point between ranges A and B, while the critical failure load is at the transition point between ranges B and C. The loading process was divided into two stages: post-compression and secondary cyclic compression. The stability behavior curve is shown in Figure 1.

Using RLT tests and analyzing a large number of studies, Werkmeister [13,14] formulated a standard method for evaluating the stability behavior of granular materials under cyclic loading. The standard method is based on the cumulative axial strain generated by 3000 to 5000 load cycles. The change rate of cumulative axial strain between two loading times can be calculated, and then the specimen state can be evaluated according to that rate. The specific evaluation criteria are as follows:

Range A: $\Delta \varepsilon_{5000}-\Delta \varepsilon_{3000}<4.5\times 10^{-5}$, the material is in the elastic stability state.
Range B: $4.5\times 10^{5}<\Delta \varepsilon_{5000}-\Delta \varepsilon_{3000}<4.5\times 10^{4}$, the material is in the plastic creep state.
Range C: $\Delta \varepsilon_{5000}-\Delta \varepsilon_{3000}>4.5\times 10^{4}$, the material is in the incremental plastic failure state.

In recent years, Chen [28] improved the standard method and verified it using the research data of Tao [29], Werkmeister [14], Perez [30], and Gu [12]. The rationality of the method was also proved. The standard method is based on the creep formula proposed by Yin [31] to describe granular materials:

(1) $$\Delta \varepsilon =\frac{\psi_{0}^{\prime }\log\left[\left(t+t_{0}\right)/t_{0}\right]}{1+\left(\psi_{0}^{\prime }/\Delta \varepsilon_{1}\right)\log\left(t+t_{0}\right)},$$

where Δε represents the cumulative creep strain, *t* refers to the number of load cycles during creep, and $\Psi_{0}^{\prime }$, *t*_0_, and $\Delta \varepsilon_{1}$ are constants.

**Figure 2:**
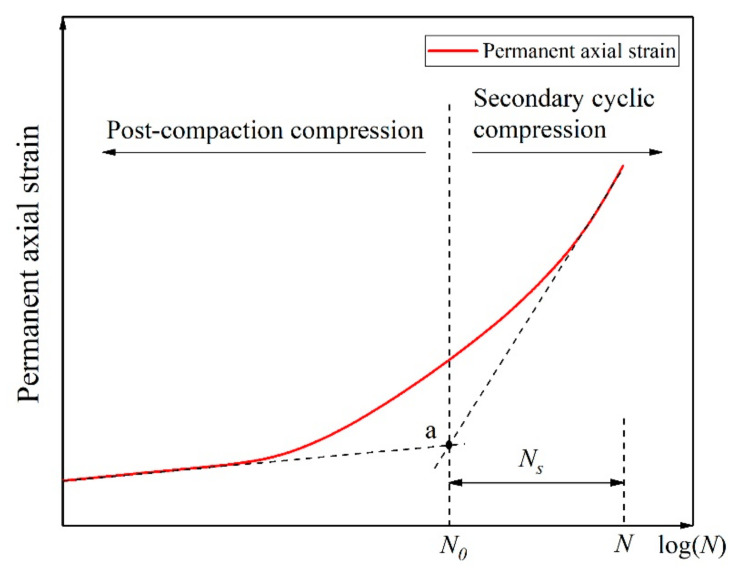
Selection of numbers of rotating axial compression cycles, reprinted with permission from [28]. Copyright 2019 Elsevier.

In this study, *t* and *t*_0_ in Equation (1) are replaced by the number of cycles *N* and *N*_0_ (Figure 2), and the constants are replaced by *n_s_* and *m_s_*, The following formula results:

(2) $$\Delta \varepsilon =\frac{\log\left[\left(N_{S}+N_{0}\right)\right]}{n_{s}+m_{s}\times \log\left[\left(N_{S}+N_{0}\right)/N_{0}\right]}.$$

**Figure 3:**
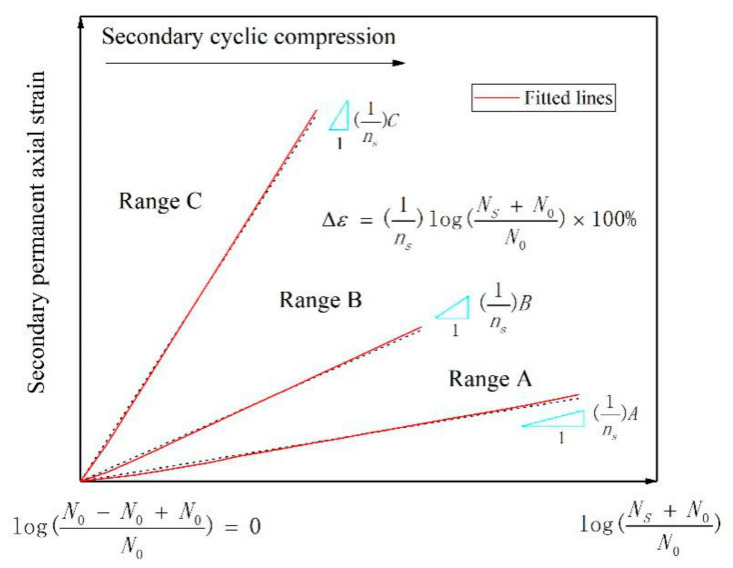
Relationship between cumulative axial strain and rotation number, reprinted with permission from [28]. Copyright 2019 Elsevier.

In Equation (2), 1/*m_s_* represents the cumulative axial strain of the specimen when *N* tends to infinity, and 1/*n_s_* represents the slope between Δε and the strain curve (Figure 3).

In Figure 2, a represents the turning point on the permanent axial strain curve, *N*_0_ represents the cycle number of the turning point on the permanent axial strain curve, *N* represents the total cycle number in a test, *N_S_* is equal to *N* minus *N*_0_.

Further, the following relationship can be obtained:

(3) $$1/n_{s}=\frac{\Delta \varepsilon }{\log\left[\left(N_{S}+N_{0}\right)/N_{0}\right]\times 100\%}$$

and 1/*n_s_* can be used to represent the rate of cumulative axial strain change and to evaluate the stability behavior of granular materials under cyclic rotating axial compression.

Based on a summary and analysis of the existing research data, Chen [28] obtained the specific numerical relationship between 1/*n_s_* and stability behavior, as follows:

Range A: $1/n_{s}\leq 0.1$, the specimen is in the elastic stability state.
Range B: $0.1<1/n_{s}\leq 0.434$, the specimen is in the plastic creep state.
Range C: $0.434<1/n_{s}$, the specimen is in the incremental plastic failure state.

Based on this method, in this research, the evaluation rules for stability behavior of the graded crushed stone specimen under cyclic rotating axial compression are proposed, and the critical loads of the specimen that indicate when the plastic creep and failure occur are obtained.

## 3. Raw Materials and Specimen Preparation

### 3.1. Raw Materials

1. Fine aggregate: crushed granite crushed stone particles with particle size below 4.75 mm used in the Guigang Expressway in Guangxi.
2. Coarse aggregate: crushed granite crushed stone particles with particle size from 4.75 to 53 mm used in the Guigang Expressway in Guangxi. The properties of the crushed stone materials were tested according to specification [32], and these properties are shown in Table 1.

### 3.2. Specimen Preparation Method

**Figure 4:**
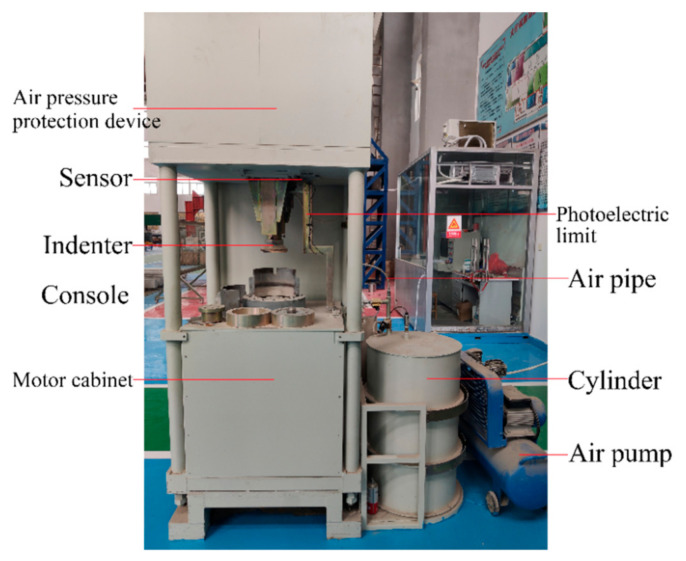
Road material vibration rotary compaction device.

The specimen molding device used in this study for the crushed stone mixture preparation was an in-house developed road material vibration rotary compaction device shown in Figure 4. As shown in Figure 4, the device uses air pressure to protect the pressurized parts, the rotation and vibration motors of the device are placed under the console, and the pressure and displacement sensors are set on the right side of the indenter. The device performs vibration, rotation, and static compaction, and the three functions can be applied individually or in combination. The specific performance parameters of the device are as follows: vibration frequency of 3000 times/min, vibration amplitude of 0.6 mm, rotation rate of 5 rpm, and static pressure of 100–700 kPa. The device can accomplish a variety of different specimen preparation methods. In order to better simulate the rolling mechanism of road rollers in the process of road construction, a preparation method for the graded crushed stone mixture specimen using vibration and rotary compaction was proposed.

The specific specimen preparation steps were as follows:

1. Take 5000 g of crushed stone mixture according to intended gradation.
2. Add water to the 5000 g crushed stone mixture according to the best moisture content of 4.1% and mix evenly. Seal with plastic and maintain for 12 h.
3. Place the crushed stone mixture in two layers and compact each layer for 4 min.

**Figure 5:**
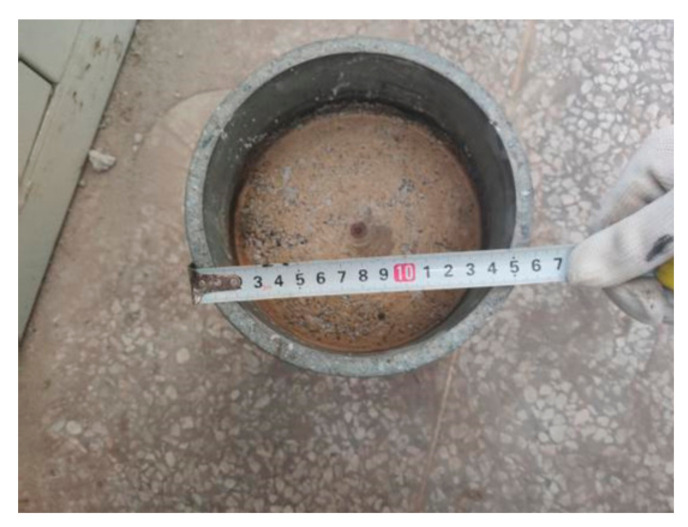
Crushed stone mixture specimen after preparation.

As shown in Figure 5, the inner diameter and height of the specimen tube were 150 and 230 mm, respectively. The height of the prepared specimens was usually between 120 and 125 mm, and the compacted density of crushed stone was between 2.3 and 2.45 g/cm^3^, which was close to the value for practical engineering applications.

## 4. Experimental Program

### 4.1. Loading Method of Cyclic Rotating Axial Compression

**Figure 6:**
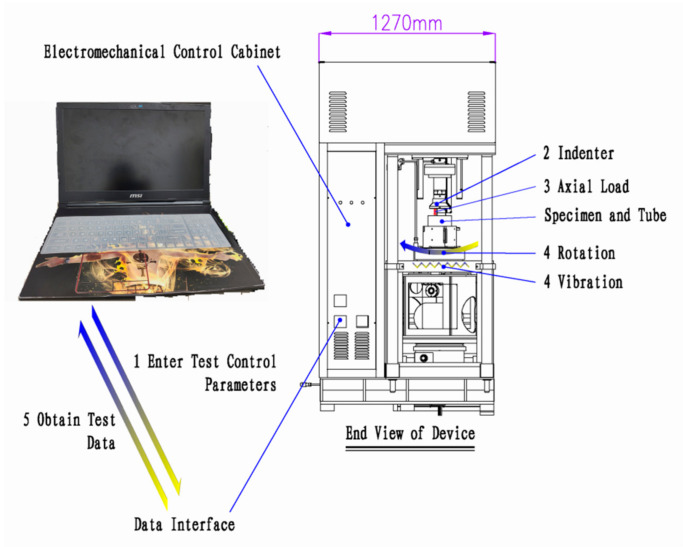
Schematic diagram of instrument loading system.

The road material vibration rotary compaction device can apply a cyclic rotating axial compression load by performing rotation and axial compression concurrently. The device and its working principles are shown in Figure 6. First, the parameters that control the operation of the device are entered using a computer connected to the device using an optical fiber cable. Then, the indenter of the device begins to drop and apply pressure when it touches the top surface of the crushed stone mixture. When the pressure reaches the set value, the rotation and vibration motor of the device starts to work. Finally, experimental data are obtained by the operating software of the device. The combined effect of axial stress and shear stress was achieved by the simultaneous application of rotation and compression actions, with the axial pressure controlled by a computer during the loading process.

**Figure 7:**
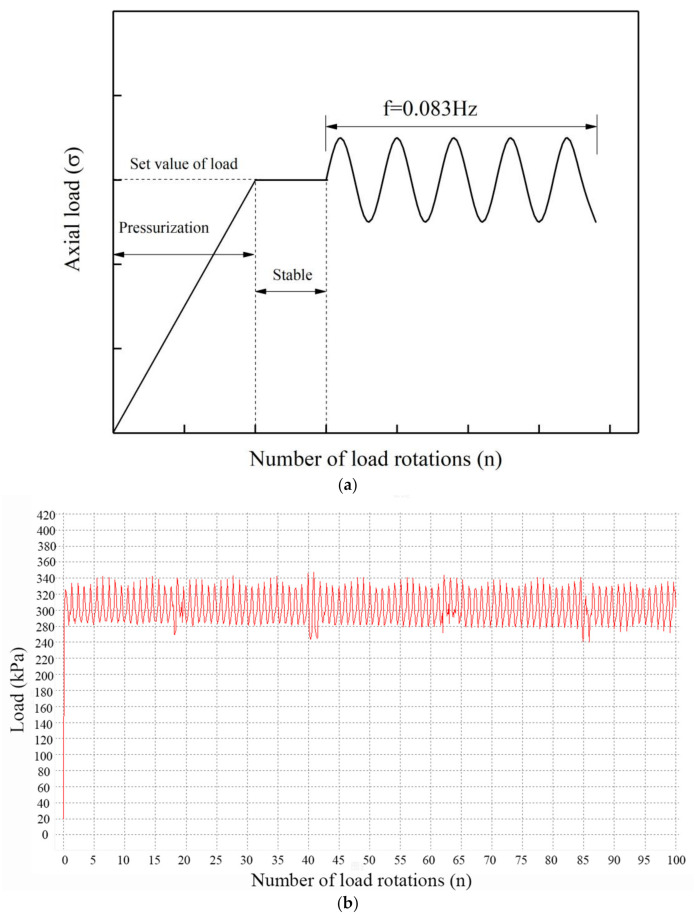
(**a**) Loading process and test load waveform; (**b**) real-time load during cyclic rotating axial compression.

The indenter of the road material vibration rotary compaction device can automatically identify the load during the test; thus, after setting the load, the device can automatically adjust the load according to the measured load values to ensure the load is stable and remains in the set range of about 20% of the set value. An example of the loading process and load waveform during testing is shown in Figure 7a.

**Figure 8:**
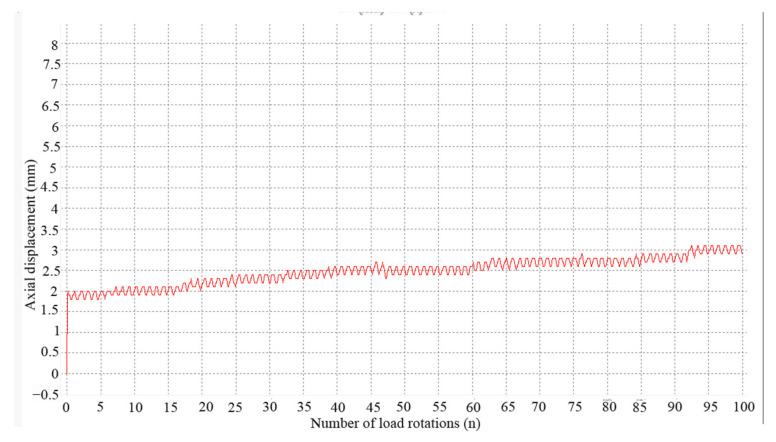
Real-time displacement under cyclic rotating axial compression.

As shown in Figure 7a, the loading process included three stages: pressurization, during which the axial load increased up to the set value for the first time; stabilization, when the axial load attained the set value and paused for a short term; and finally, the stage when the axial load fluctuated. Figure 7b shows the changing load values during the loading process. Figure 8 shows the axial displacement of a specimen during loading in the road material vibration rotary compaction device.

In this study, a road material vibration rotary compaction device was used to apply cyclic rotating axial pressure on large-size graded crushed stone specimens. According to the authors of [28], the experiments can be terminated when the deformation trends of specimens become clear under repeated loading, therefore, 100 load rotations were adopted (20 min). In order to test the shakedown behavior and calculate the critical load of large-size crushed stone specimens under cyclic loading, experimental displacement results need to be obtained from specimens subjected to different load levels [28], therefore, the axial load was divided into seven levels. The cumulative axial strain of the graded crushed stone specimens under the different levels of cyclic rotating axial pressure was collected by a computer.

The shakedown theory was used to evaluate the deformation behavior and calculate the critical shakedown and critical failure loads of the graded crushed stone—first, according to Equation (3) to calculate values of 1/*n_s_* and evaluate the shakedown behavior of specimens under different loads based on evaluation critical and second, according to Equations (4) and (5) to calculate the critical load of different gradation crushed stone specimens under cyclic loading. Finally, the influence of different factors on the shakedown behavior and the stability of the specimen under cyclic loading were analyzed.

### 4.2. Influence of Gradation on Stability

**Figure 9:**
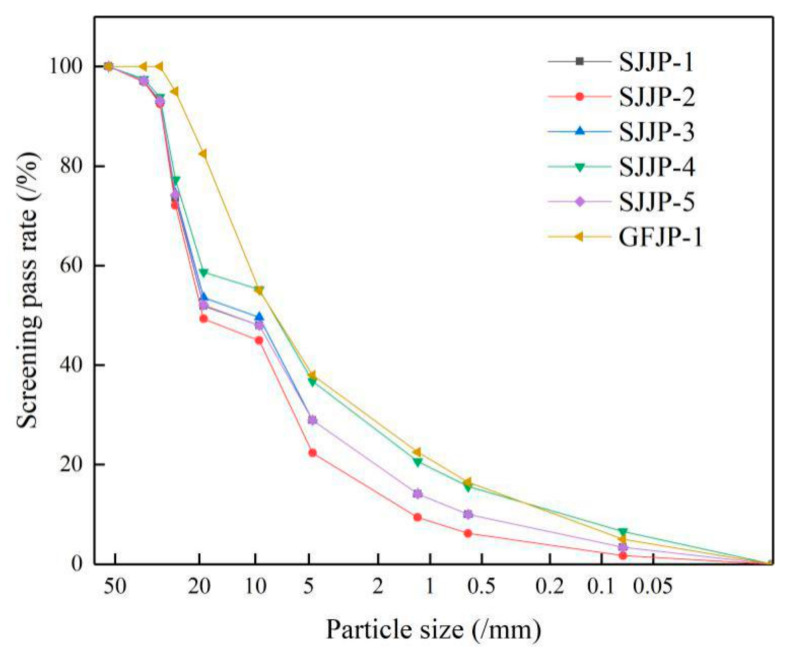
Gradation graph for all gradations.

The step-by-step filling method [21] was used to design the three-level coarse aggregate ratio, and the I method [22] to design the fine aggregate ratio. Then five groups with three samples per group of large-size graded crushed stone gradations to be subjected to a single load level were obtained by mixing coarse and fine aggregates, these type of gradations are named as DG-1 to 5 (Design Gradation) in Table 2. The median values of the upper and lower limits of the design ranges were calculated according to the gradation design ranges recommended by the specification [32]. The median value of the specification was taken as the gradation of the conventional-size crushed stone mixture, and this type of gradation is named as SG-1 (Standard Gradation) in Table 2. The gradation graph of SG-1 and DG-1 to 5 are shows in Figure 9. In this research, three specimens of each gradation type of crushed stone were used for testing the deformation behavior under each load level, and the average displacement values of three specimens under every five cycles of cyclic loading were calculated for the subsequent analysis. Hence, this study used a total of 126 specimens to test the deformation behavior of crushed stone mixtures under cyclic loading. Finally, the stability of large-size graded crushed stone and conventional-size graded crushed stone under cyclic rotating axial compression was analyzed and compared. 

### 4.3. Influence of Skeleton Structure Performance on Long-Term Stability Based on CBR Values

The CBR values of six groups of graded crushed stone were measured. This study used five specimens of each crushed stone gradation type for testing the CBR values, and the average CBR values of five specimens were calculated for the subsequent analysis. Hence, this study obtained 30 CBR values for crushed stone. The CBR value is an important parameter influencing the local load bearing capacity and skeleton structure properties of granular materials. By analyzing the relationship between the CBR value and the cumulative axial strain and critical load of the specimen under cyclic rotating axial compression, the influence of the skeleton structure performance on the specimen stability was studied.

## 5. Analysis of Test Results

### 5.1. Deformation Relationship for Large-Size Graded Crushed Stone under Cyclic Rotating Axial Compression

**Figure 10:**
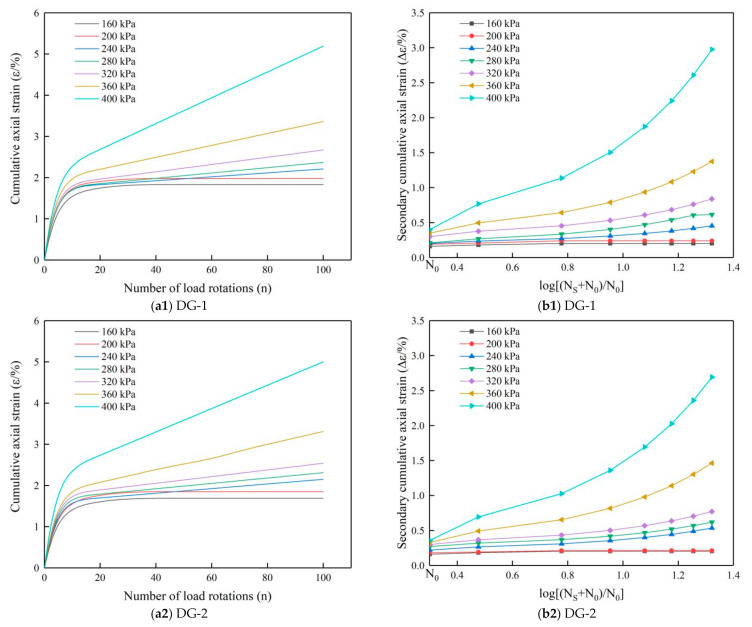

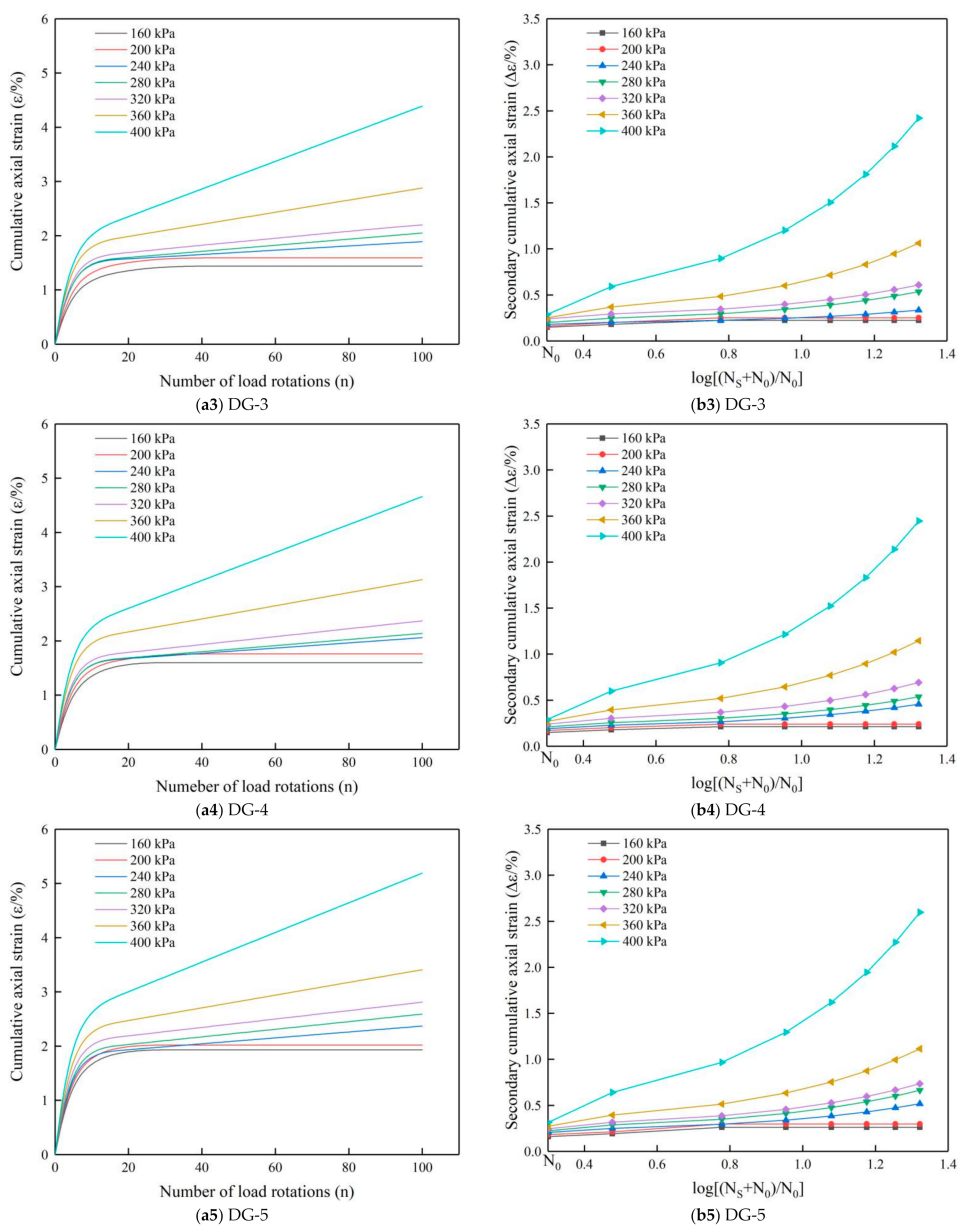
(**a1**–**a5**) Cumulative axial strain of large-size graded crushed stone of five gradation types under different loads; (**b1**–**b5**) secondary cumulative axial strain versus log[(*N_S_* + *N*_0_)/*N*_0_] of large-size graded crushed stone of five gradation types under different loads.

**Figure 11:**
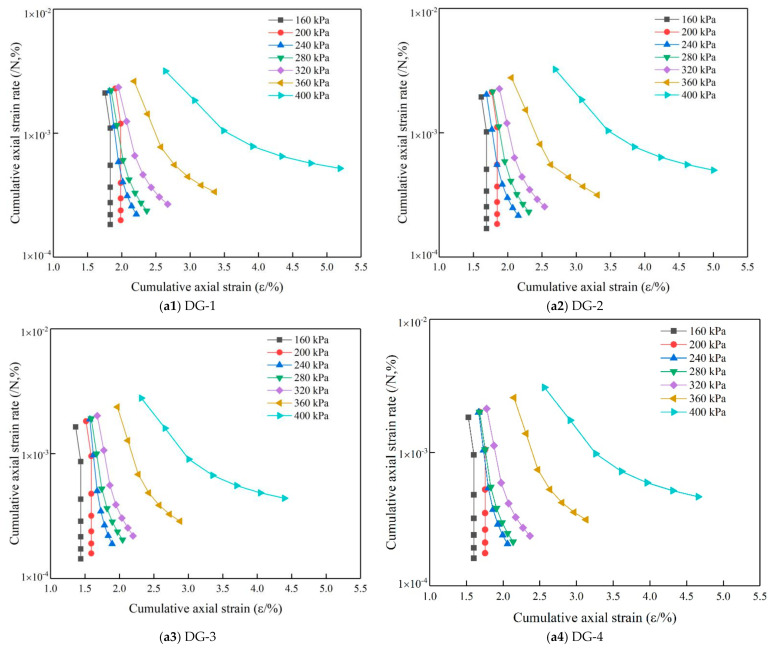

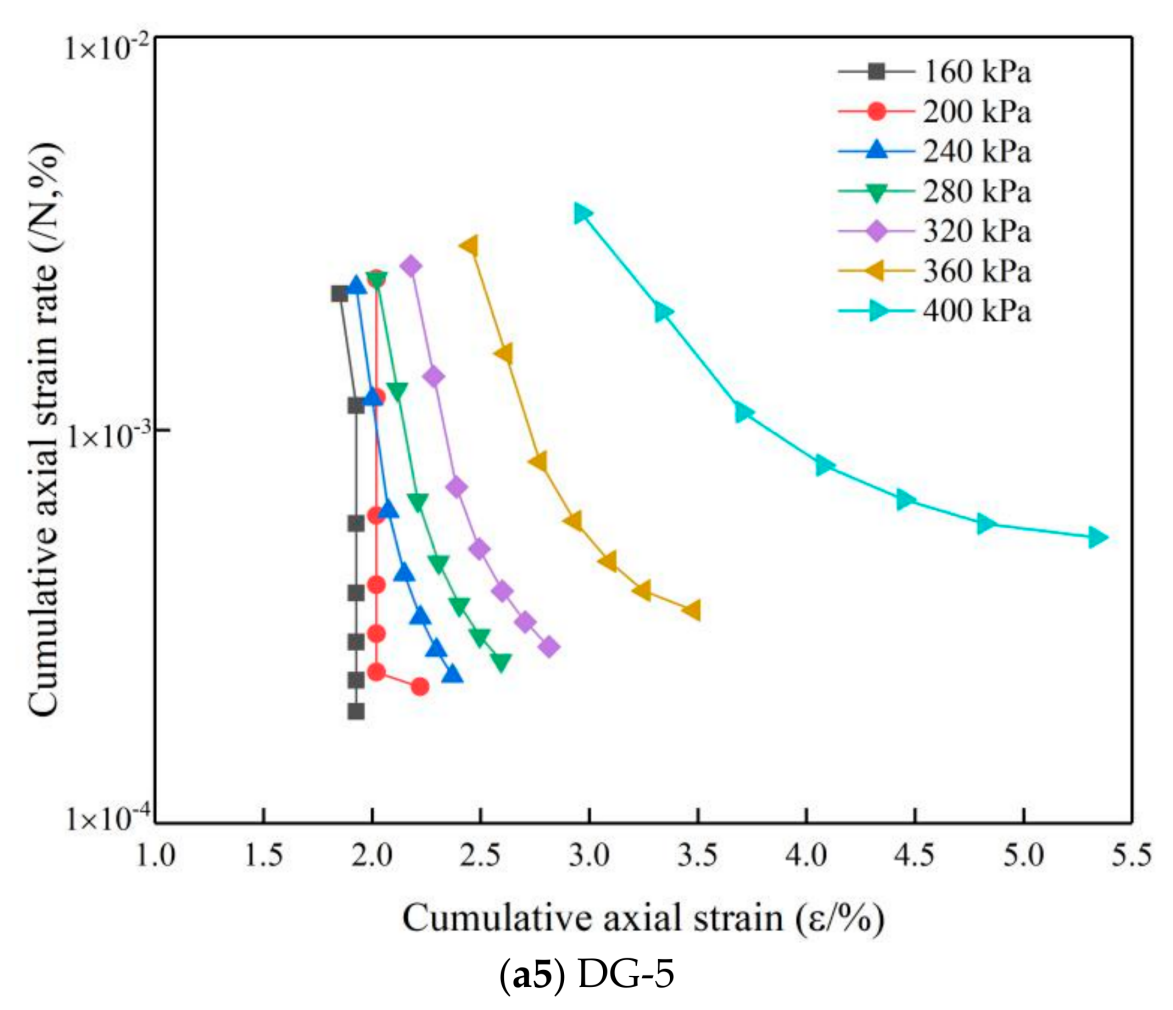
Axial strain change rate. (**a1**) DG-1; (**a2**) DG-2; (**a3**) DG-3; (**a4**) DG-4; (**a5**) DG-5.

The deformation trends of graded crushed stone became clear after 100 cycles of compression, and the experiments were terminated at 100 load cycles (20 min). The relationship between the cumulative axial strain and the number of load rotations for the large-size graded crushed stone after 100 cycles of cyclic axial compression with different load levels is shown in Figure 10, and the cumulative axial strain rate curve of all types of specimens is shown in Figure 11.

As shown in Figure 10a, the test results indicate that the deformation relationships for the large-size graded crushed stone specimens under cyclic rotating axial compression differ at different load levels. When the load was 160 and 200 kPa, the cumulative axial strain of the specimens did not increase after the elastic deformation stage, and the specimens were essentially in the stable elastic state. However, when the load was between 240 and 320 kPa, the specimens first went through the rapid strain accumulation stage of the elastic deformation stage, but subsequently, the cumulative axial strain increased only slightly with the increase of the number of load rotations. Then, when the load reached 360 kPa the cumulative axial strain of the specimens still increased rapidly after the elastic deformation stage. Finally, when the load was 400 kPa the cumulative axial strain change rate became very high, and it is assumed that the skeleton structure of the specimens had been destroyed, which clearly signifies the incremental plastic failure state.

Figure 10(a1–a5) shows that the deformation behavior of graded crushed stone under cyclic loading can be divided into two distinct stages. In the first stage (0 < *N* < *N*_0_), the specimens mainly underwent elastic deformations due to post-compaction by cyclic loading; in the second stage (*N*_0_ < *N*), the specimens mainly showed plastic deformations under the secondary cyclic compression. Figure 10(b1–b5) shows the relationship between secondary cumulative axial strain and log[(*N_S_* + *N*_0_)/*N*_0_], and demonstrates that the secondary cumulative axial strain was proportional to log[(*N_S_* + *N*_0_)/*N*_0_] when *N*_0_ < *N*.

Figure 11 shows the variation of the axial strain rate of the specimens under cyclic loading. The test results show that the strain rate of the specimens decreased sharply with the increase in the number of load rotations under different levels of cyclic axial compression load, and with the increase of the load level, the cumulative axial strain and change rate of the axial strain also increased.

**Figure 12:**
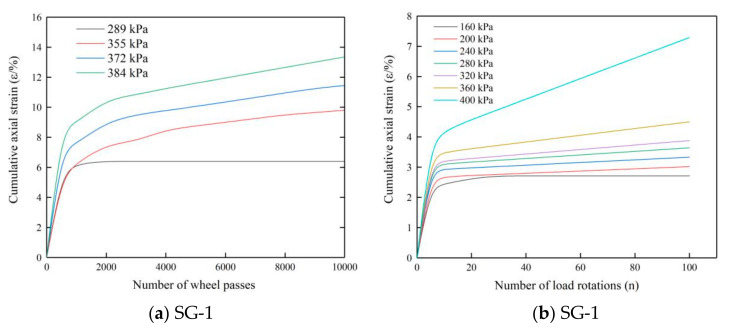
(**a**) Deformation relationship for graded crushed stone mixture under cycling wheel load; (**b**) deformation relationship for graded crushed stone mixture under cyclic rotating axial compression.

In reference [22], the stability of graded crushed stone used in highway base under cyclic loading was explored experimentally. The cyclic loading used in this study simulated wheel rolling, and the specific deformation relationship for the graded crushed stone (SG-1) under cyclic loading is shown in Figure 12a. Figure 12b shows the deformation relationship for the conventional-size graded crushed stone (SG-1) under cyclic rotating axial compression. The deformation trends in the two diagrams are essentially the same. Under cyclic loading, the graded crushed stone first underwent elastic deformations and then tended to stabilize or continue to accumulate plastic deformations.

The axial strain of graded crushed stone under a cycling wheel rolling load was larger than that under a cyclic rotating axial pressure, because the former is an unconfined test, while the latter is a confined test in a steel tube. Based on the comparative analysis of the test data in [19,33], the final cumulative axial strain of granular materials under cyclic loading was between 1% and 15%. Therefore, the cyclic rotational axial compression method proposed in this paper is valid for testing the stability of graded crushed stone and can correctly capture the deformation relationship for the graded crushed stone under cyclic loading.

### 5.2. Influence of Gradation on Shakedown Stability

#### 5.2.1. Stability Analysis of Specimens with Different Gradations

The shakedown theory is used to evaluate the deformation relationship for graded crushed stone specimens under different load levels. According to Equation (3), to calculate values of 1/*n_s_* (*N*_0_ = 5, *N* = 100, and *N_S_* = *N* – *N*_0_), the calculation results as listed in Table 3. The specific evaluation method evaluates the deformation behavior of graded crushed stone specimens under cyclic rotating axial compression by calculating the slope of the strain curve, 1/*n_s_*, as mentioned in Section 2.2. The evaluation criteria are as follows:

Range A: $1/n_{s}\leq 0.1$, the specimen is in the elastic stability state.
Range B: $0.1<1/n_{s}\leq 0.434$, the specimen is in the plastic creep state.
Range C: $0.434<1/n_{s}$, the specimen is in the incremental plastic failure state.

The results of the calculations and evaluation of shakedown behavior are shown in Table 3. According to the relationship between the 1/*n_s_* values and load level, the specific values of critical loads were calculated by interpolation. The formula for calculating the critical shakedown load is as follows:

(4) $$F_{a}=\frac{0.1-(\frac{1}{n_{s1}})}{\left(\frac{1}{n_{s2}})-(\frac{1}{n_{s1}}\right)}\times 40+F_{a1},$$

where *F_a_* represents the critical shakedown load, 1/*n_s_*_1_ indicates that the value of the shakedown behavior parameter, 1/*n_s_*, is less than 0.1 but close to 0.1, 1/*n_s_*_2_ indicates that the value of 1/*n_s_* is greater than 0.1 but close to 0.1, and *F_a_*_1_ represents the test load value corresponding to 1/*n_s_*_1_.

The formula for critical failure load is as follows:

(5) $$F_{p}=\frac{0.434-(\frac{1}{n_{s3}})}{\left(\frac{1}{n_{s4}})-(\frac{1}{n_{s3}}\right)}\times 40+F_{p1},$$

where *F_p_* represents the critical failure load, 1/*n_s_*_3_ indicates that the value of the shakedown behavior parameter, 1/*n_s_*, is less than 0.434 but close to 0.434, 1/*n_s_*_4_ indicates that the value of 1/*n_s_* is greater than 0.434 but close to 0.434, and *F_p_*_1_ represents the test load value corresponding to 1/*n_s_*_3_.

The calculation results are shown in Table 4 and demonstrate that the critical shakedown load of the conventional-size graded crushed stone specimens is more than 10% lower than that of the large-size graded crushed stone specimens, while there is little difference in the critical failure load between the two types of specimens. When the specimens are subjected to a lower cyclic rotating axial compression, the main source of deformation is the rearrangement of particles caused by particles slipping inside the graded crushed stone mixture. When the cyclic rotating axial load increases to the value of the critical failure load, the large-size crushed stones will be broken, and the relative slip between the particles will form the overall plastic deformation.

Previous studies [34,35,36] indicate that the deformation caused by the overall rearrangement of the particles generated by the slip in graded crushed stone mixtures was mainly related to their density, gradation characteristics, and voids. Therefore, specimens with a higher density and stronger skeleton structure exhibited smaller slip deformations under the same load. It can be observed that the skeleton structure performance in SJJP-3 and SJJP-4 is better than in the other specimens. In the graded crushed stone specimen, the relative slip deformation between the particles caused by the crushing of large-size crushed stone is mainly related to the crushing and compaction condition, which does not differ significantly under the same load [37,38].

#### 5.2.2. Effect of Content and Density of Large-Size Crushed Stone on Critical Load

The particle size of large-size crushed stone is larger than 26.5 mm. The relationship between the large-size crushed stone content, the critical shakedown load, and the specimen density is shown in Figure 12. The data of large-size crushed stone content and density are shown in Table 5, and the effect of large-size crushed stone content on the long-term stability is analyzed.

**Figure 13:**
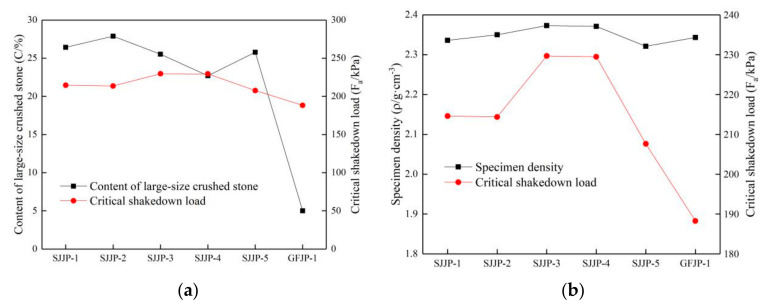
(**a**) Relationship between critical shakedown load and content of large-size crushed stone; (**b**) relationship between critical shakedown load and specimen density.

As shown in Figure 13, the critical shakedown load of the specimen is not only related to the large-size crushed stone content but also related to the density of the specimen; thus, a joint analysis can better describe this relationship. The lowest content of large-size crushed stone in the conventional-size graded crushed stone (GFJP-1) was 5%, much lower than that in the other five groups. The critical shakedown load was also the lowest, as shown in Figure 13a. The content of large-size crushed stone in the five groups of large-size graded crushed stone specimens (SJJP-1 to 5) ranged between 22% and 28%, and the relationship between the large-size crushed stone content and its critical shakedown load was not clear. According to the relationship between the density specimens and the critical shakedown load in Figure 13b, the critical shakedown load increased with the increase in the density. The large-size crushed stone content of SJJP-3 was 25.52% and that of SJJP-4 was 22.70%. The density of these two groups was the highest among the six groups of specimens, and the critical shakedown loads were larger than those of the other groups of specimens. Therefore, for the crushed stone mixture specimen, when the content of large-size crushed stone was between 22% and 28%, and the critical shakedown load increased with the increase in density.

**Figure 14:**
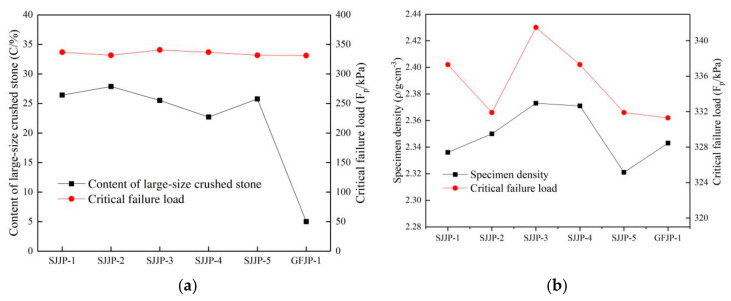
(**a**) Relationship between critical failure load and content of large-size crushed stone; (**b**) relationship between critical failure load and specimen density.

The relationships between the content of large-size crushed stone, the critical failure load, and the specimen density are shown in Figure 14. It is noted that the critical failure load of the specimens under cyclic loading was not strongly related to the content and density of large particle-size crushed stone. The reason is that the deformation of large-size graded crushed stone is mainly related to the stone crushing condition under a high level of cyclic rotating axial compression as mentioned in Section 4.2, and the stone crushing condition under the same load was almost the same.

#### 5.2.3. Effect of Content and Density of Large-Size Crushed Stone on Cumulative Axial Strain

**Figure 15:**
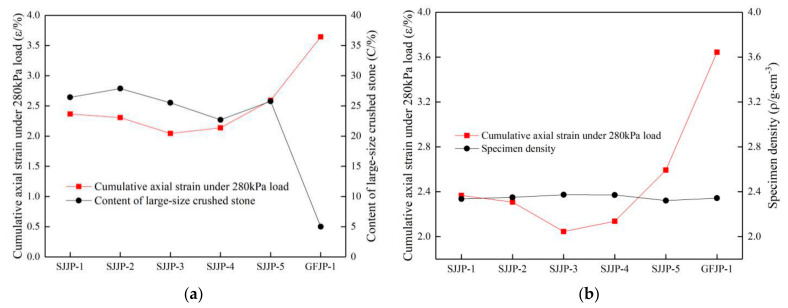
(**a**) Relationship between cumulative axial strain and content of large-size crushed stone content under load of 280 kPa; (**b**) relationship between cumulative axial strain and specimen density under load of 280 kPa.

The relationship between the content and density of large-size crushed stone and the cumulative axial strain under the 280 kPa load is shown in Figure 15. It is noted that the large-size crushed stone content of GFJP-1 was 5%, which is lower than those of other specimens, and the cumulative axial strain was the highest. The specimens with a lower large-size crushed stone content had low stiffness. In the five groups of the large-size graded crushed stone specimens (SJJP-1 to 5), the content of large-size crushed stone was between 22% and 28%. The cumulative axial strain of specimens was not closely related to the large-size crushed stone content but rather to the density of specimen. The cumulative axial strain decreased with the increase in specimen density. The large-size crushed stone content of SJJP-3 was 25.52%, while in SJJP-4 it was 22.70%. The densities of the two specimens were similar and higher than those of the other four specimens, but the cumulative axial strains of the two specimens were smaller than those of the other specimens. It can be seen that when there was little difference in the content of large-size crushed stone, the main factor affecting the specimen stiffness was the density of the specimen. Therefore, when the content of large-size crushed stone was between 22% and 28%, the cumulative axial strain decreased with the increase in the specimen density.

Based on the above analysis, it can be concluded that the large-size graded crushed stone has better stiffness and load bearing capacity than the conventional-size graded crushed stone. When the content of the large-size crushed stone is appropriate, the higher the specimen density is, the larger the stiffness and load bearing capacity is. In the six groups of graded crushed stone specimens, the stiffness and load bearing capacity of SJJP-3 and SJJP-4 were better than those of the other specimens, thus the best gradation design range of large-size crushed stone is between those of SJJP-3 and SJJP-4.

#### 5.2.4. Regression Analysis of the Joint Effect of Content and Density of Large-Size Crushed Stone

**Figure 16:**
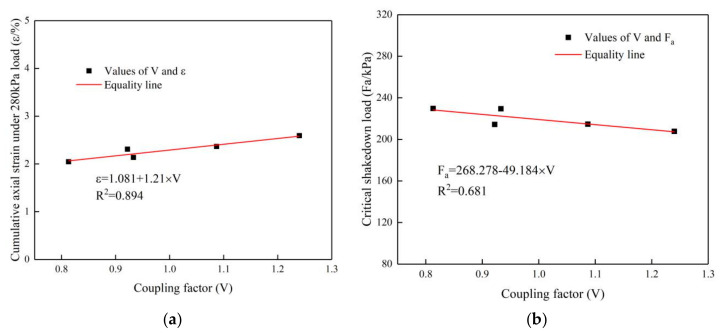
(**a**) Regression analysis between coupling factor and cumulative axial strain under a cyclic rotating axial compression of 280 kPa; (**b**) regression analysis between coupling factor and critical shakedown load.

The joint effect of the large-size crushed stone content and density on the cumulative axial strain and critical shakedown load of DG-1 to 5 samples under a cyclic rotating axial compression of 280 kPa were analyzed. The coupling factor was used to quantify the joint effect of large-size crushed stone content and density, and the specific coupling factor formula as follows:

(6) $$V=\frac{\rho -2.4693}{-0.0464\times C}\times 10,$$

where *V* is the coupling factor, *ρ* is the specimen density (g/cm^3^), and *C* is the content of large-size crushed stone (%). The coupling factors of large-size crushed stone content and density of DG-1 to 5 were calculated, and the linear regression analysis was carried out between the coupling factors and the cumulative axial strain and critical shakedown load under a cyclic rotating axial compression of 280 kPa. The results are shown in Figure 16. It was found that there is a good linear relationship between the large-size crushed stone content and density coupling factor of DG-1 and the cumulative axial strain and critical shakedown load under a cyclic rotating axial compression of 280 kPa. The analysis demonstrated that the joint effect of large-size crushed stone’s content and density on its mechanical properties and anti-deformation ability is strong.

### 5.3. Relationship between CBR Values, Strain, and Critical Load

#### 5.3.1. Relationship between CBR Values and Cumulative Axial Strain of Specimen

**Figure 17:**
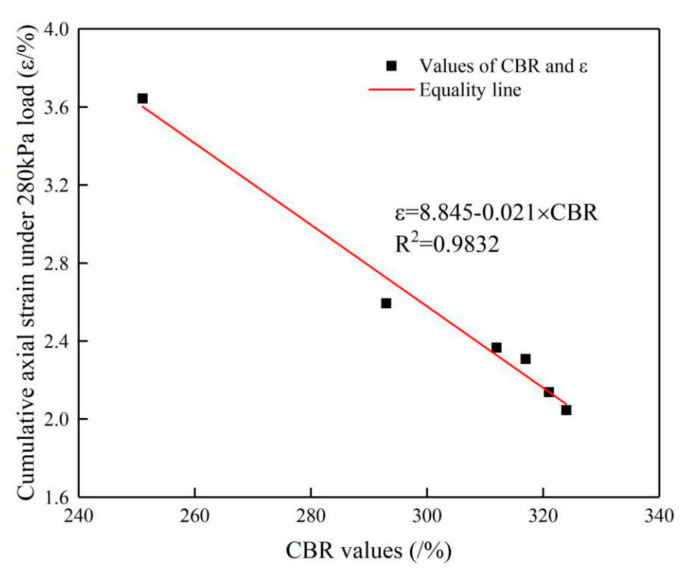
Relationship between CBR values and cumulative axial strain under a 280 kPa load.

The curves in Figure 17 show that the cumulative axial strain of the specimens with low CBR values is larger than that of other specimens under the same level load, which indicates that the specimens with weaker skeleton structure have weaker stiffness and therefore develop larger deformation under the same load level. Moreover, the CBR values of the conventional-size graded crushed stone are smaller than those of the large-size graded crushed stone, and the cumulative axial strains of the conventional-size graded crushed stone are also larger than those of the large-size graded crushed stone. Thus, the large-size graded crushed stone has better skeleton structure properties than the conventional-size graded crushed stone.

#### 5.3.2. Relationship between CBR Values and Critical Load

**Figure 18:**
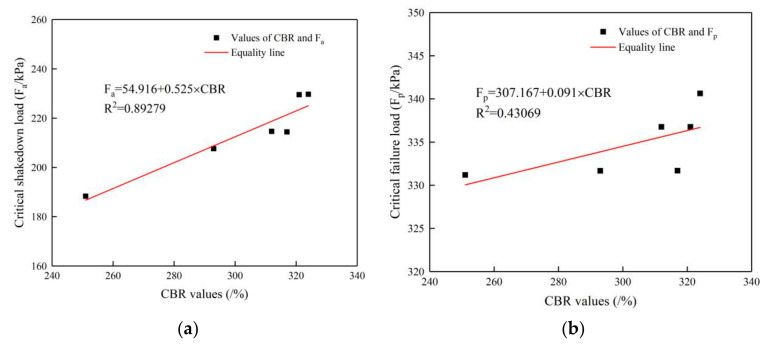
(**a**) Relationship between CBR values and critical shakedown load; (**b**) relationship between CBR values and critical failure load.

The curves in Figure 18a show that the CBR values are closely related to the specimen’s critical shakedown load under cyclic loading. The critical shakedown load increases with an increase in the CBR value of the specimen, which indicates that the skeleton structure of large-size graded crushed stone strongly influences its stiffness, and the stronger the skeleton structure is, the larger the stiffness is. The correlation between the CBR values and the critical failure load of the specimen in Figure 18b is not as clear as that in Figure 18a, because there is little difference in the stone crushing condition under the same loads.

From the above analyses of the test results, it can be concluded that there is a strong correlation between the CBR values of the large-size graded crushed stone and its critical shakedown load under cyclic loading. The critical shakedown load increases with the increase in the CBR value, while there is no strong correlation between the CBR value and the critical failure load. The reason is that the failure resistance of the specimen is mainly related to the properties of the crushed stone but not to the skeleton structure performance.

## 6. Conclusions

This research investigated the deformation behavior of granular materials under cyclic loading using the traditional RLT tester and the MTS tester. The deformation behavior of large-size graded crushed stone was tested under the simultaneous action of axial pressure and shear stress using the road material vibration rotary compaction device. Different gradations of the selected materials were tested in order to evaluate the effect of the content of large-size crushed stone and density. This study also introduced a new method to calculate the critical load of granular materials under cyclic loading based on shakedown theory. Some of the main conclusions can be summarized as follows:

1. In the first part of the research, the cumulative axial strain of 126 crushed stone specimens under cyclic loading was analyzed. The cumulative axial strain of large-size graded crushed stone specimen will eventually become stable and no longer increase under a cyclic rotating axial compression of 200 kPa. When the load is between 240 and 320 kPa, the cumulative axial strain of large-size graded crushed stone will still increase slowly after elastic deformations, but the strain rate will decrease rapidly. When the load is increased to 360 kPa, the cumulative axial strain of the large-size graded crushed stone increases rapidly during the entire process of cyclic loading, and the strain rate is maintained at a large value. It is observed that the deformation relationship and strain values of the specimens under cyclic rotating axial compression are similar to those in existing literature. Therefore, it is feasible to apply cyclic rotating axial compression to study the long-term stability of large-size graded crushed stone used in road base.
2. In the second part, the critical load of graded crushed stone specimens under cyclic loading was calculated based on the shakedown theory and the new calculation method. The maximum critical shakedown load of large-size graded crushed stone was 229.655 kPa and the lowest was 207.619 kPa, respectively. The critical shakedown load of the conventional-size graded crushed stone was 188.276 kPa, making the critical shakedown load of the large-size graded crushed stone at least 10% higher than that of the conventional-size graded crushed stone. This indicates that the large-size graded crushed stone can be used in engineering applications, and it deforms less. The analysis clearly showed that the content of the large-size crushed stone, density, and skeleton structure performance influence the critical shakedown load. On the contrary, there is no significant difference between the critical failure load of the large-size graded crushed stone and that of the conventional-size graded crushed stone, with both being between 330 and 345 kPa.
3. In the third part, the relationship between the cumulative axial strain and gradation of graded crushed stone was analyzed. When the content of large-size graded crushed stone with a size over 26.5 mm was between 22% and 28%, the higher the density was, and the larger the stiffness, the better the skeleton structure performance and the load bearing capacity were. Therefore, the content of the large-size crushed stone in the large-size graded crushed stone mixture should be controlled between 22% and 26%, and the density should be as high as possible.
4. In addition, the skeleton structure performance of crushed stone influences the stability behavior of crushed stone specimens under cyclic loading was observed. The CBR values of 30 specimens and the deformation data of 126 specimens under cyclic loading test showed that there is a linear relationship between CBR values and cumulative axial strain and critical shakedown load of crushed stone specimens. The linear correlation coefficient between CBR values and critical shakedown load was 0.89279 and cumulative axial strain of specimens under a 280 kPa load cyclic loading was 0.9832, respectively.
5. In short, this study proposed the method is using the vibration and rotary compaction method for preparation of specimens of crushed stone and testing their stability under cyclic rotating axial compression in the road material vibration rotatory compaction device. The method achieved simultaneous testing under axial pressure and shear stress using the device. The test data of 126 crushed stone specimens show that this is a simple and practical method for testing the stability of granular materials under cyclic loading, and it enables analyzing the correlations between deformation behavior and gradation parameters. Therefore, this method could be used for investigating the stability of granular materials under a cycling load.

## Figures and Tables

**Table 1:** Technical specifications of aggregates.

Aggregate (mm)	Crushing Value (%)	Apparent Density (g/cm^3^)	Dry Density (g/cm^3^)	Gross Relative Volumetric Density	Water Absorption Rate (%)	Needle Flake Content (%)
19–53	21.57	2.729	2.672	2.646	1.24	7.30
9.5–19	14.08	2.753	2.691	2.662	1.32	6.58
4.75–9.5		2.735	2.702	2.684	0.70	6.89
0–4.75		2.720	2.705	2.694	0.92	
Specification requirements	≤28	Measured data	Measured data	Measured data	Measured data	≤15

**Table 2:** Screening pass rate of each gradation.

Gradation Group	Mass Percentage Passing through Screen Hole (mm)/%									
	53	37.5	31.5	26.5	19	9.5	4.75	1.18	0.6	0.075
DG-1	100	97.05	92.87	73.57	51.93	48.00	28.92	14.13	9.98	3.43
DG-2	100	96.89	92.47	72.12	49.29	44.96	22.35	9.41	6.18	1.70
DG-3	100	97.15	93.11	74.48	53.58	49.62	28.92	14.13	9.98	3.43
DG-4	100	97.47	93.87	77.30	58.71	55.19	36.77	20.63	15.59	6.58
DG-5	100	97.15	93.10	74.23	52.09	47.98	28.92	14.13	9.98	3.43
SG-1	100	100	100	95.00	82.50	55.00	38.00	22.50	16.50	5.00

**Table 3:** Values of 1/*n_s_* value and shakedown behavior of graded crushed stone under different loads.

Gradation Group	1/*n_s_* (160 kPa)	Behavior	1/*n_s_* (200 kPa)	Behavior	1/*n_s_* (240 kPa)	Behavior	1/*n_s_* (280 kPa)	Behavior	1/*n_s_* (320 kPa)	Behavior	1/*n_s_* (360 kPa)	Behavior	1/*n_s_* (400 kPa)	Behavior
DG-1	0.058	A	0.058	A	0.173	B	0.289	B	0.289	B	0.635	C	1.847	C
DG-2	0.058	A	0.058	A	0.175	B	0.291	B	0.349	B	0.640	C	1.803	C
DG-3	0.057	A	0.057	A	0.115	B	0.229	B	0.286	B	0.573	C	1.604	C
DG-4	0.058	A	0.058	A	0.115	B	0.173	B	0.289	B	0.635	C	1.617	C
DG-5	0.056	A	0.084	A	0.168	B	0.280	B	0.336	B	0.672	C	1.793	C
SG-1	0.059	A	0.117	B	0.117	B	0.235	B	0.352	B	0.645	C	2.462	C

**Table 4:** Critical loads of six gradation groups of graded crushed stone.

Gradation Group	Critical Shakedown Load (kPa)	Critical Failure Load (kPa)
DG-1	214.609	336.763
DG-2	214.359	331.684
DG-3	229.655	340.627
DG-4	229.474	336.763
DG-5	207.619	331.667
SG-1	188.276	331.195

**Table 5:** Contents and densities of large-size crushed stone specimens.

Gradation Group	Content of Large-Size Crushed Stone with Size over 26.5 mm (%)	Density (g/cm^3^)
DG-1	26.43	2.336
DG-2	27.88	2.350
DG-3	25.52	2.373
DG-4	22.70	2.371
DG-5	25.77	2.321
SG-1	5.00	2.343

## Data Availability

Data sharing is not applicable to this article.
